# Ablation of Akt2 Induces Autophagy through Cell Cycle Arrest, the Downregulation of p70S6K, and the Deregulation of Mitochondria in MDA-MB231 Cells

**DOI:** 10.1371/journal.pone.0014614

**Published:** 2011-01-31

**Authors:** Stacey A. Santi, Hoyun Lee

**Affiliations:** 1 Tumor Biology Group, Regional Cancer Program of the Sudbury Regional Hospital, Sudbury, Ontario, Canada; 2 Department of Biochemistry, Microbiology and Immunology, University of Ottawa Medical School, Ottawa, Ontario, Canada; 3 Division of Medical Sciences, Northern Ontario School of Medicine, Sudbury, Ontario, Canada; Yale Medical School, United States of America

## Abstract

**Background:**

Akt/PKB is a promising anticancer therapeutic target, since abnormally elevated Akt activity is directly correlated to tumor development, progression, poor prognosis and resistance to cancer therapies. Currently, the unique role of each Akt isoform and their relevance to human breast cancer are poorly understood.

**Methodology/Principal Findings:**

We previously found that Akt1, 2 and 3 are localized at specific subcellular compartments (the cytoplasm, mitochondria and nucleus, respectively), raising the possibility that each isoform may have unique functions and employ different regulation mechanisms. By systematically studying Akt-ablated MDA-MB231 breast cancer cells with isoform-specific siRNA, we here show that Akt2 is the most relevant isoform to cell proliferation and survival in our cancer model. Prolonged ablation of Akt2 with siRNA resulted in cell-cycle arrest in G0/G1 by downregulating Cdk2 and cyclin D, and upregulating p27. The analysis of the Akt downstream signaling pathways suggested that Akt2 specifically targets and activates the p70S6K signaling pathway. We also found that Akt2 ablation initially resulted in an increase in the mitochondrial volume concomitantly with the upregulation of PGC-1α, a regulator of mitochondrial biogenesis. Prolonged ablation of Akt2, but not Akt1 or Akt3, eventually led to cell death by autophagy of the mitochondria (i.e., mitophagy).

**Conclusions/Significance:**

Collectively, our data demonstrates that Akt2 augments cell proliferation by facilitating cell cycle progression through the upregulation of the cell cycle engine, and protects a cell from pathological autophagy by modulating mitochondrial homeostasis. Our data, thus, raises the possibility that Akt2 can be an effective anticancer target for the control of (breast) cancer.

## Introduction

Akt serine/threonine kinase plays a crucial role in the regulation of cell growth, proliferation, and survival. Constitutive activation of the Akt pathway is associated with tumor development, poor prognosis, and resistance to anticancer agents [1]. All three known Akt isoforms (Akt1–3) show a high degree of sequence similarity, and contain three functional domains: the N-terminal PH domain, the catalytic domain in the middle, and the C-terminus [2], [3]. Mainly due to technical difficulties, many published works do not delineate Akt functions with each isoform. Therefore, some of the known Akt functions may be applicable to one isoform but not others, although the three Akt isoforms apparently share some functional redundancy [4], [5].

The availability of isoform-specific antibodies and siRNAs allowed us to study the unique function of each Akt isoform. Using these tools, we have recently found that Akt1–3 are localized mainly to the cytoplasm, the mitochondria, and the nucleus, respectively, in many cancer cell lines including cervical, breast, and prostate cancer cells [6]. This specific subcellular localization may ultimately underlie the regulation of isoform-specific functions. Thus, it was hypothesized that a certain Akt isoform may be more important for cell survival than the other isoforms [7].

To gain a further understanding, we examined the specific role played by each Akt isoform in the regulation of cell proliferation and survival using the MDA-MB231 breast cancer model. The isoforms (singularly or in combination) were ablated by selectively targeting each isoform with isoform-specific siRNA. Cell survival, cell proliferation, and cell cycle progression over a prolonged period (0–120 h post-transfection) were examined. Downstream substrates known to be regulated by Akt were also examined to determine isoform-specific pathways. Our data shows that Akt2 is most relevant to cell proliferation and survival among the three Akt isoforms. Akt2 ablation resulted in the upregulation of p27^Kip1^ (p27), and the downregulation of Cdk2 and cyclin D, leading to cell cycle arrest in G0/G1. Data obtained from the study of the downstream Akt pathways suggests that Akt2 specifically regulates the phosphorylation of p70S6K, a downstream target of mTOR. We also found that the prolonged inactivation of Akt2 caused an abnormal increase in mitochondrial volume and, eventually, led to autophagy of the mitochondria (i.e., mitophagy). Collectively, our data demonstrates that Akt2 is crucial for cell proliferation and survival by facilitating cell cycle progression, augmenting p70S6K activity, and modulating mitochondrial biogenesis.

## Results

### Akt2 ablation resulted in a substantial decrease in cell proliferation

**Figure 1:**
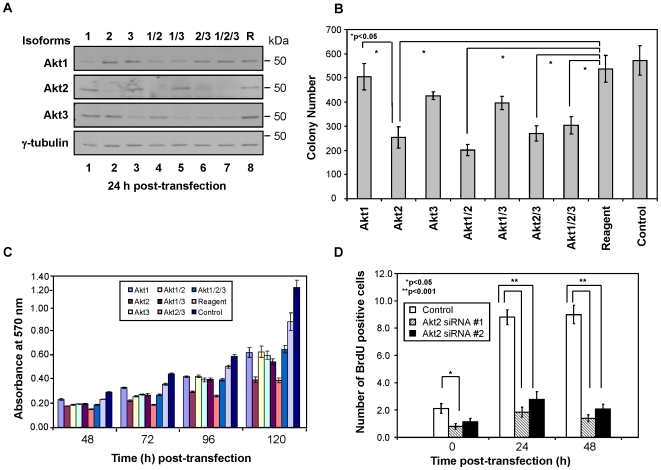
Akt2 ablation resulted in a substantial decrease in cell proliferation and cell survival. (**A**) The isoform-specific siRNA oligos used in this study effectively downregulated protein synthesis in an isoform-specific manner. Western blotting was carried out with samples isolated from MDA-MB231 cells transfected with isoform-specific siRNA alone or in combination with other siRNA oligos as indicated. “R” denotes cells transfected with reagent alone (i.e., a mock transfection control). Akt1–3 at the left of the panel denotes antibodies against Akt1–3 proteins, respectively. γ-tubulin was used as a loading control. Data shown is representative of four independent experiments using siRNA set #1. (**B**) Among three Akt isoforms, Akt2 ablation caused the greatest decrease in the colony forming ability. Results shown have employed siRNA set #1. Data shown is an average of three independent experiments. Error bars are standard errors. (**C**) Data from an MTT assay showed that ablation of Akt2 downregulated cell proliferation. MDA-MB231 cell proliferation was analyzed at 48–120 h post-transfection using siRNA set #2. Data shown is representative of four independent experiments. (**D**) DNA replication is dramatically downregulated in MDA-MB231 cells transfected with Akt2 siRNA. Cells were pulse-labelled with BrdU for 45 min, followed by analysis of DNA replication at 0–48 h post-transfection with an anti-BrdU antibody. Both siRNA oligos to Akt2 (#1 and #2) show reduced cell proliferation relative to the control (p<0.05). Data is expressed as mean BrdU incorporation, and error bars are standard errors. Results shown are an average of three independent trials.

To study the unique role of each Akt isoform, siRNA oligonucleotides were transfected into MDA-MB231 cells. This cell line was chosen as a breast cancer model system, as we had previously shown the subcellular localization of the Akt isoforms to be consistent among MDA-MB231, MCF7, and MDA-MB468 breast cancer cell lines [8]. Given that MDA-MB231 cells are p53 negative and ER negative, the use of this cell line allowed us to examine the unique role of the Akt isoforms in an aggressive model of breast cancer. In addition, MDA-MB231 cells exhibit no known alterations in the Akt pathway. As shown in Fig. 1A and Fig. S1A, the Akt siRNA oligos used in our experiments effectively downregulated the Akt proteins in an isoform-specific manner. Isoform specificity of the oligonucleotides was confirmed by Western blotting in these experiments and no cross-reactivity was observed even when one Akt isoform-specific siRNA was used in combination with other siRNA(s) (i.e., knockdown of Akt1 did not knockdown Akt2 or Akt3) (Fig. 1A, lanes 1–7). The downregulation of each Akt isoform was routinely achieved at the range of 40–70% with both sets of isoform-specific siRNA oligos, which usually coincided with the transfection efficiency, suggesting that these isoform-specific siRNA oligos are highly effective.

To determine the role of each Akt isoform in cell survival, we carried out a clonogenic assay. MDA-MB231 cells were transfected with either set of two isoform-specific siRNA oligos, alone or in combination with other isoform-specific oligo(s), followed by colony formation analysis at 12–14 days post-transfection. The Akt2 knockdown caused the most significant decrease in the colony forming ability among the three Akt isoforms (p<0.05) (Fig. 1B). Colony-formation assays performed with the other set of siRNA oligos showed a similar result (Fig. S1B).

To gain insight into the possible mechanism of how Akt2 may promote cell survival, we examined cell proliferation in the Akt knockdowns. Akt has previously been established as a critical regulator of cell cycle progression (reviewed in [9]). However, there are conflicting reports as to which Akt isoform is involved in the regulation of cell cycle progression [10]–[15]. To determine if the reduced colony formation observed in the Akt2-ablated cells was due to a decrease in cell proliferation, MTT assays were carried out with the cells transfected with isoform-specific siRNA oligos at 48–120 h post-transfection. Data obtained from the experiment demonstrated that knockdown of any Akt isoform resulted in a decrease of cell proliferation by 120 h post-transfection. However, the decrease in cell proliferation was most pronounced with cells transfected with Akt2 siRNA (Fig. 1C). Reduced cellular proliferation was also observed in the double knockdown when Akt2 was one of the isoforms involved (i.e., Akt1/2 and/or Akt2/3); however, the double knockdown did not show synergistic effects in cell proliferation. The relatively high survival rate of the Akt1, 2 and 3 triple knockdown sample is probably due to the low transfection efficiency often shown when cells are transfected with all three isoform-specific siRNA oligos. Alternatively, it is also possible that certain Akt isoforms may (directly or indirectly) function antagonistically each other with respect to cell proliferation and survival.

To further confirm the MTT data, DNA replication activities were examined by analyzing incorporation of BrdU in cells transfected with Akt2 siRNA. The results showed that the incorporation of BrdU into DNA was dramatically decreased in the cells transfected with Akt2 siRNA (p<0.001) (Fig. 1D). It should be noted that cell proliferation was substantially reduced both in siRNA-treated and scrambled siRNA-treated samples at the 0 h timepoint. This is because cells were incubated in serum-free medium for 24 h during the transfection procedure. (Note that 0 h is defined as the end point of the 24-h transfection period). When cells were in full serum for 24–48 h post-transfection, BrdU-positive staining was observed only in the control samples, confirming that Akt2-ablated cells were not proliferating (p<0.001) (Fig. 1D). Data obtained with the Akt2 siRNA set #2 yielded a similar result (Fig. 1D).

### Cdk2 is downregulated in the Akt2-ablated cells

We analyzed cell cycle progression by flow cytometry since cell proliferation, survival, and DNA replication were downregulated in the Akt2-ablated cells. Cells were transfected with Akt2 siRNA in serum-free medium for 24 h. At the end of transfection (i.e., 0 h timepoint), the medium was changed to complete medium containing 10% FBS. The cell cycle profiles for the controls and Akt2 siRNA-treated samples were similar at the 0 h timepoint, which were consistent with those arrested at G0/G1 by serum starvation (Fig. S2). Cells in the “Reagent” and “Kept-in-incubator” controls progressed into the cell cycle by 24 h post-transfection in the complete medium (Fig. S2). In contrast, cells treated with Akt2 siRNA did not progress into the cell cycle at the same timepoint (Fig. S2). Our data suggests that the decrease in cell number observed in both MTT and clonogenic assays was not due to an increase in cell death, at least by 48 h post-transfection, since there was no substantial number of Akt2-ablated cells with sub-G1 DNA content (Fig. S2).

**Figure 2 pone-0014614-g002:**
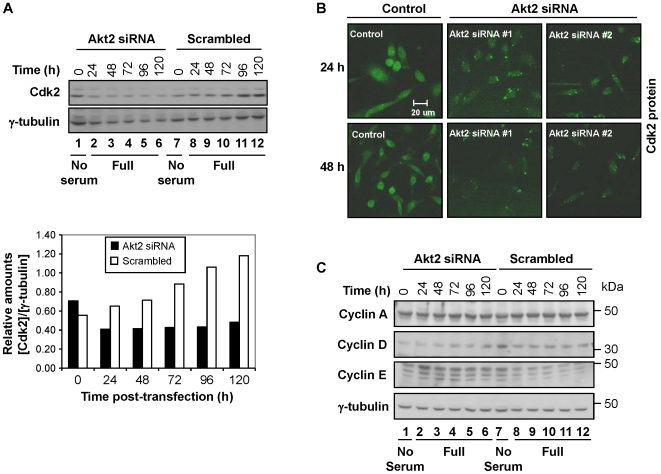
Cdk2 and cyclin D were downregulated in cells transfected with Akt2-siRNA. (**A**) The relative level of Cdk2 was measured in a time course manner (0–120 h post-transfection) after cells were transfected with Akt2 siRNA (set #2). (Lower panel) The relative intensity of bands shown in the upper panel was normalized with the loading control and presented in a graph format. “No serum” denotes Opti-MEM transfection medium described in Materials and Methods. “Full” denotes culture medium containing 10% FBS (i.e., complete medium). (**B**) Analysis of Cdk2 by immunostaining in cells transfected with Akt2 siRNA (either oligo #1 or #2). The sampling times were 24 and 48 h post-transfection. Images shown are representative of three independent trials for each timepoint and each siRNA, respectively. (**C**) Akt2 ablation resulted in the downregulation of cyclin D, but not cyclin A and E. Western blot analysis of cyclin A, D and E of the cells transfected with Akt2 siRNA or scrambled siRNA was carried out at 0–120 h post-transfection. Note that the 0 h timepoint is incubated for 24 h in serum-free medium for both Akt2-siRNA and scrambled-siRNA treated cells. Data shown is representative of three independent experiments.

To further investigate the mechanism of the cell cycle arrest in the Akt2-ablated cells, we examined the levels of several proteins involved in cell cycle control. We first chose Cdk2 as a primary candidate, since published data suggested that Akt is involved in the regulation of Cdk2 in an isoform-specific manner [16], [17]. As shown in Fig. S3A (upper and lower panels), the downregulation of Cdk2 was obvious in the Akt2-ablated cells, but not in the Akt1- and Akt3-ablated cells. We found that the level of Cdk2 was low throughout the entire period when Akt2-ablated cells were maintained in complete medium (i.e., 24–120 h post-transfection) (Fig. 2A, upper and lower panels; Fig. S3B). The degree of Cdk2 downregulation was generally greater in the later timepoints after Akt2 ablation. The downregulation of Cdk2 in the Akt2-ablated cells was confirmed by indirect immunostaining with an anti-Cdk2 antibody (Fig. 2B).

We also examined the levels of cyclins A, D and E in the cells treated with Akt2 isoform-specific siRNA. Cyclin D was downregulated in Akt2-ablated cells, which is consistent with the cell cycle arrest in G0/G1 in the Akt2-ablated cells (Fig. 2C; Fig. S3C). Unexpectedly, the level of cyclin E was upregulated in theAkt2-ablated cells (Fig. 2C; Fig. S3D). It was previously shown that Cyclin E is degraded by the ubiquitin pathway through SCF-Fbw-ubiquitin ligase and subsequent binding to Skp1 (reviewed in [18]). The degradation of cyclin E is prevented when Cdk2-cyclin E complexes are inhibited by p27, a Cdk inhibitor [19]. Thus, Cdk2-p27 binding can inhibit the turnover of cyclin E. We found that the level of cyclin A was not altered by Akt2 ablation (Fig. 2C). It should be noted that cyclin D was also downregulated in Akt1-ablated cells, but not in Akt3-ablated cells (Fig. S4).

### p27 was upregulated and localized in the nucleus of Akt2-ablated cells

The finding that Akt2-ablated cells are arrested in G0/G1 prompted us to examine the level of p27, as its high level in the nucleus is directly relevant to the cell cycle arrest at G0/G1. Furthermore, p27 is also a known PI3K/Akt target, as the inhibition of this signaling pathway with LY294002, wortmannin or rapamycin resulted in an increase of p27 protein [20]–[23]. However, it is currently unknown which Akt isoform is involved in the regulation of p27 protein.

The level of p27 may be regulated by transcription and/or protein degradation which is mediated by the post-translational modification-ubiquitination pathway [24]–[26]. Nuclear p27 may bind to the Cdk2-cyclin D cell cycle engine to inactivate its kinase activity, resulting in cell cycle arrest at G0/G1. The Akt-mediated regulation of p27 could be through FoxO transcription factors [27], mTOR [28], and/or direct phosphorylation of p27 at Thr157 [29]–[31] and Thr198 [32]. Phosphorylated p27 by Akt is translocated to the cytoplasm, where it is subjected to ubiquitin-mediated proteasomal degradation by Skp2. This process is required for cell cycle progression to occur (reviewed in [33]). p27 is also phosphorylated by Cdk2-cyclin E kinase at Thr187 during S phase, which also becomes the target of Skp2-mediated protein degradation (reviewed in [34]).

**Figure 3 pone-0014614-g003:**
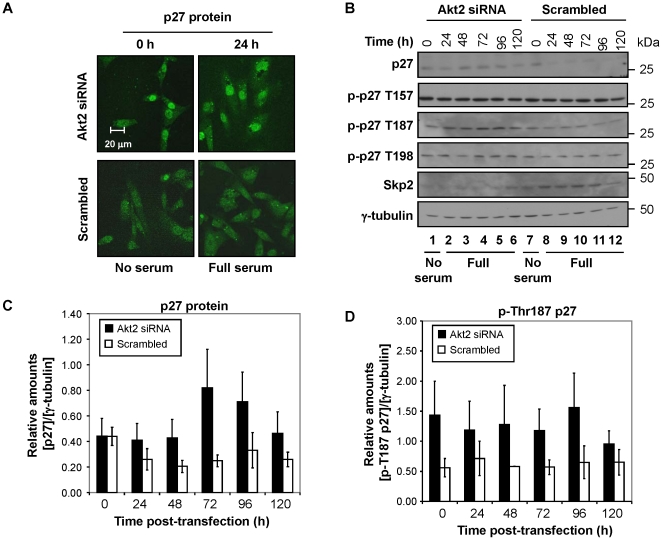
p27 was localized to the nucleus and its level increased in Akt2-ablated cells. (**A**) Data from immunostaining assays shows that p27 was mainly localized to the nucleus of the cells transfected with Akt2 siRNA. Confocal images shown are representative of three independent experiments. (**B**) Akt2-ablated cells showed an increase in the level of p27 but a decrease in Skp2 proteins. The relative levels of total (**C**) and phosphorylated p27 proteins at T187 (**D**) are shown.

We found that p27 was localized to the nucleus of Akt2-ablated cells, but a low level of p27 was ubiquitously present throughout the entire cell in the control (Fig. 3A). (It should be noted that transfection started and continued for 24 h prior to the 0 h timepoint.) In addition, data from Western blotting showed that the level of p27 protein was substantially higher in the cells transfected with Akt2 siRNA than the control (Fig. 3B, C). Together, our data suggests that both the nuclear localization and the elevated level of p27 play important roles in the G0/G1 cell cycle arrest by Akt2 ablation.

If the nuclear localization of p27 was mediated through its phosphorylation by Akt, the level of phosphorylated p27 (particularly on the Thr157 residue) would be lower in the Akt2-ablated cells than in the scrambled control. Surprisingly, both Akt-mediated phosphorylation sites (Thr157 and Thr 198) were not significantly altered by the Akt2-ablation (Fig. 3B; Fig. S5A, B). However, phosphorylation at Thr187 was upregulated in the Akt2-ablated cells, compared to the control (Fig. 3B, D). This could be due to the decrease in the Skp2-mediated degradation of phosphorylated p27 at Thr187 in the Akt2-ablated cells, as the level of Skp2 was markedly downregulated in Akt2-ablated cells across all the timepoints examined (Fig. 3B).

Since the regulation of p27 in the Akt2-transfected cells was not mediated by the Thr157 and Thr198 Akt-mediated phosphorylation, we examined the level of p27 mRNA by quantitative PCR. As expected, cells maintained in serum-free medium for 24 h (i.e., the duration of the entire siRNA transfection) contained a high level of p27 mRNA, regardless of the Akt2 status (Fig. S5C, 0 h). Once cells were released into complete medium after the transfection procedure ended, the level of p27 mRNA was lower both in the control and Akt2-ablated cells by 24 h post-transfection; however, the relative level was much lower in the Akt2-ablated cells than the control (Fig. S5C). This result was surprising since the p27 protein level was higher in Akt2-ablated cells than the control at the same timepoint (Fig. 3B, lines 2 and 8; Fig. 3C, 24 h). This data may suggest that p27 protein was more stable upon release into complete medium, and this could potentially be the result of the downregulation of Skp2 at the same timepoint (Fig. 3B). The level of p27 mRNA in Akt2-ablated cells became more abundant by 48 h, followed by a peak at 72 h post-transfection (Fig. S5C, D). Collectively, the level of p27 mRNA generally follows that of p27 protein in the Akt2-ablated cells, with the exception of the 24 h post-transfection timepoint. Unlike the Akt2-ablated cells, p27 protein was not significantly altered by the ablation of Akt1 or Akt3 (Fig. S6).

### Akt2 ablation markedly downregulated the phosphorylation of p70S6K at Thr389

Akt regulates cell proliferation and survival through the activation and/or inactivation of many downstream substrates, including GSK-3β [35] and Forkhead (FoxO) transcription factors [36]. mTOR and TSC2 (tuberin, an upstream regulator of mTOR) also regulates cell cycle progression through the upregulation and stabilization of p27 [37]–[39], in addition to their function in protein synthesis through p70S6K and 4E-BP1. We determined whether these Akt downstream proteins are regulated in an Akt2 isoform-specific manner. Data obtained from Western blotting showed that neither the level of total protein nor phosphorylation of FoxO1 at Ser256 was notably altered by Akt2 ablation (Fig. S7A), suggesting that the upregulation of p27 transcription in Akt2-ablated cells is independent of the FoxO1 signaling pathway. Similarly, neither the level of GSK-3β protein nor its phosphorylation status at Ser9 and Ser21 was substantially altered in Akt2-ablated cells (Fig. S7B).

To ensure that the decrease in cell proliferation by Akt2 ablation was not due to the downregulation of the MAPK pathway, protein levels of p-ERK1/2 (p42/44) were also examined. We found that the phosphorylation pattern of ERK1/2 (p42/44) was not substantially different between cells transfected with Akt2 siRNA or scrambled siRNA (Fig. S7B). Together, these data suggest that Akt2 is not involved in the regulation of ERK, GSK-3β, or FoxO1 signaling pathways in the MDA-MB231 cell line.

**Figure 4:**
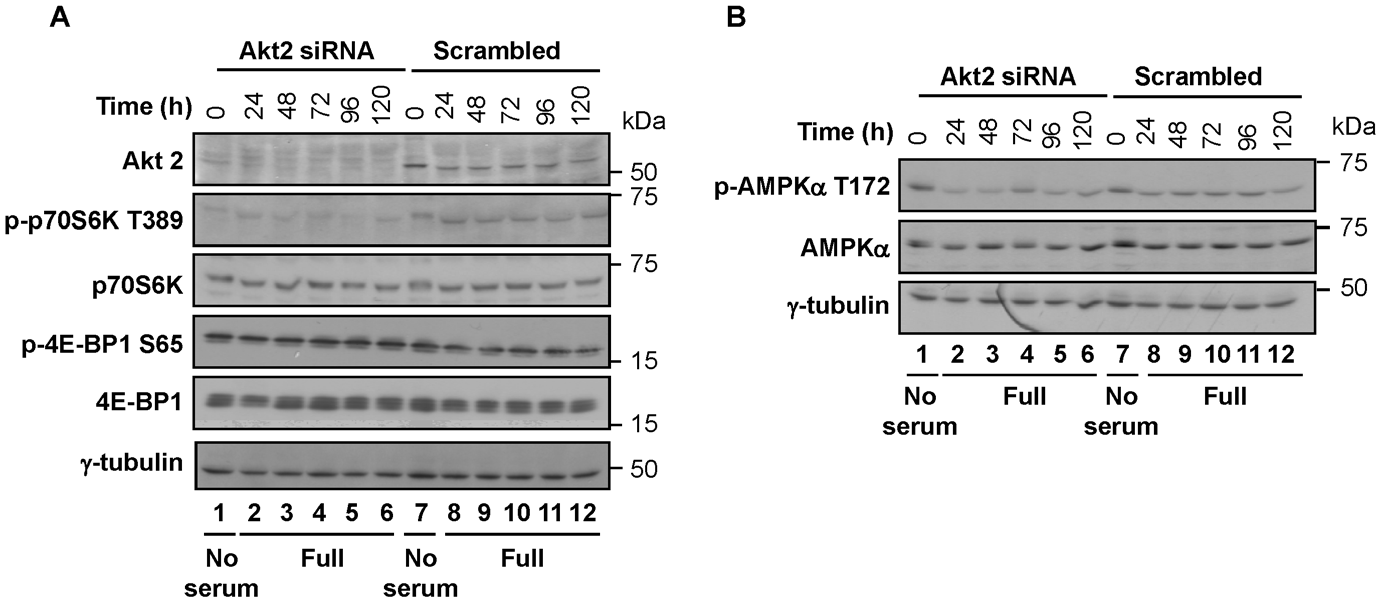
Ablation of Akt2 resulted in the downregulation of phosphorylated p70S6K and AMPKα. (**A**) Akt2 ablation caused the downregulation of p70S6K phosphorylation at T389. The activation of the mTOR pathway was analyzed by Western blotting using extracts from MDA-MB231 cells transfected with Akt2 siRNA or scrambled siRNA. The numbers 0–120 on the top of the panel are the sampling timepoints (h) after transfection. (**B**) Akt2 ablation slightly downregulated AMPKα. Note that the highest levels of p-AMPKα occur in both the Akt2-ablated and scrambled controls at the 0 h timepoint (i.e., during the serum starvation period) while the levels are lower in the Akt2-ablated cells in the later timepoints (48–120 h; i.e., maintained in complete medium). Western blot shown for p-AMPKα was replicated four times, while AMPKα was replicated in triplicate.

Since Akt is known to regulate cell cycle progression through the mTOR signaling pathway, the activities of the two branches of the mTOR pathway, p70S6K and 4E-BP1 (eukaryotic initiation factor 4E-binding protein 1) were examined. As shown in Fig. 4A, the phosphorylation of p70S6K at Thr389 was almost completely downregulated in the cells transfected with Akt2 siRNA, although the level of p70S6K protein was not significantly altered by the ablation. Unlike p70S6K, however, neither the level of protein nor the phosphorylation status of 4E-BP1 was substantially altered in the Akt2-ablated cells (Fig. 4A). Collectively, these data demonstrate that Akt2 is involved in the regulation of cell proliferation and protein synthesis through the mTOR-p70S6K signaling pathway in MDA-MB231 cells. In contrast to Akt2-ablated cells, the phosphorylation status of p70S6K was not substantially altered by either Akt1 or Akt3 ablation (Fig. S6).

AMP-activated protein kinase (AMPK), which is responsive to cellular energy depletion, can inhibit cell growth through TSC2-dependent suppression of mTOR signaling [40], [41]. Data presented by Hahn-Windgassen et al. [42] supports that Akt functions upstream of AMPKα. It is also known that AMPKα phosphorylates TSC2, thereby, constraining mTOR activity [43]. To determine whether the downregulation of the mTOR-p70S6K pathway through Akt2 ablation was mediated by the activation of AMPKα, we examined the relation between AMPKα and Akt2 ablation. Data in Fig. 4B shows that the levels of both AMPKα protein and phosphorylation at Thr172 were moderately downregulated in Akt2-ablated cells maintained in complete medium. It should be noted that AMPKα was activated (i.e., phosphorylated at Thr172) by serum deprivation (i.e., during the 24 h transfection period), regardless of the Akt2 status (Fig. 4B, lanes 1, 7).

### Mitochondrial volume was increased in Akt2-ablated cells

**Figure 5:**
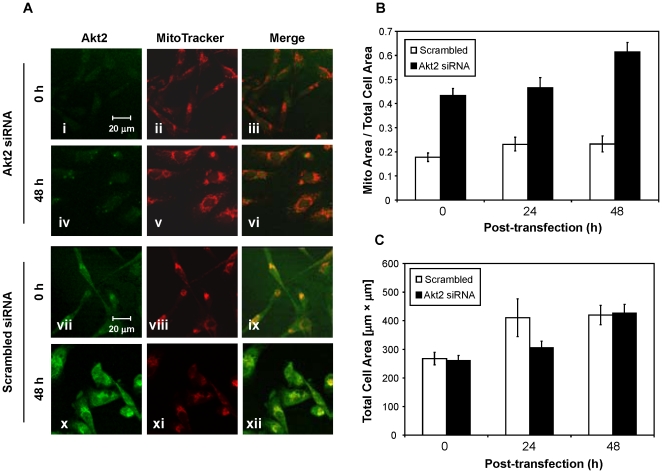
(A) Akt2 ablation caused an increase in the mitochondrial volume. Cells transfected with either Akt2 siRNA (i–vi) or scrambled siRNA (vii–xii) were immunostained with anti-Akt2 antibodies (Akt2) (green) or MitoTracker (red). Samples were taken at 0 or 48 h post-transfection as indicated. It should be noted that Akt2 is co-localized with mitochondria (ix, xii) as described previously [102]. Cells at 0 h were in the Opti-MEM (serum-free) for 24 h during transfection, and those at 48 h were maintained in the complete medium containing 10% FBS for 48 h after the transfection procedure was complete. Note that the 20 µm size bar of the scrambled samples is 1.3 fold bigger than that of Akt2-treated samples. (**B**) The relative surface area of the total mitochondria was compared to the total surface area of the cell (µm^2^). The quantification of the cells surface area was carried out using software installed in the LSM 510 Meta laser scanning confocal microscope (Carl Zeiss). For the quantification, at least ten fields were analyzed for each sample. Error bars are standard errors. (**C**) The same as panel (**B**), except this data shows total cell area (µm^2^).

It was previously shown that Akt2 was physically associated with the mitochondria in a variety of cancer cell lines including MDA-MB231 [44]. Therefore, the mitochondrial status of the Akt2-ablated cells was examined in conjunction with MitoTracker Red CMXRos, a mitochondrial membrane potential-dependent dye that is incorporated into actively respiring cells. The mitochondria in MDA-MB231 cells normally present in one of the following two morphologies: (i) aggregates around the perinuclear region; or (ii) rounded punctuate structures in the cytoplasm (Fig. 5A, panels viii, xi). However, the mitochondria in Akt2-ablated cells were spread more widely in the cytoplasm, and the overall volume was substantially increased (Fig. 5A, compared panels v and xi). A computer-assisted analysis of the mitochondrial surface area showed that the total area of the mitochondria in Akt2-ablated cells was approximately 2-fold greater than that of the control (p<0.001) (Fig. 5B). The total area of the entire cell surface was not notably altered by Akt2 ablation (Fig. 5C), suggesting that the increase in the mitochondrial surface area in Akt2-ablated cells was not due to an increase in cell size (p>0.05, ns).

### Peroxisome proliferator-activated receptor γ coactivator-1α (PGC-1α) was upregulated in Akt2-ablated cells

The increase in the mitochondrial volume in Akt2-ablated cells could be due to an increase in mitochondrial biogenesis, which is regulated by components of the AMPK, FoxO, and nitric oxide pathways [45]–[47]. mTOR has also been demonstrated to directly regulate mitochondrial biogenesis, or through the upstream AMPK signaling pathway [48], [49]. Alterations in any of these pathways may result in the inhibition or activation of PGC-1α. Thus, PGC-1α is the central player for mitochondrial biogenesis and gluconeogenesis [50]–[52].

**Figure 6:**
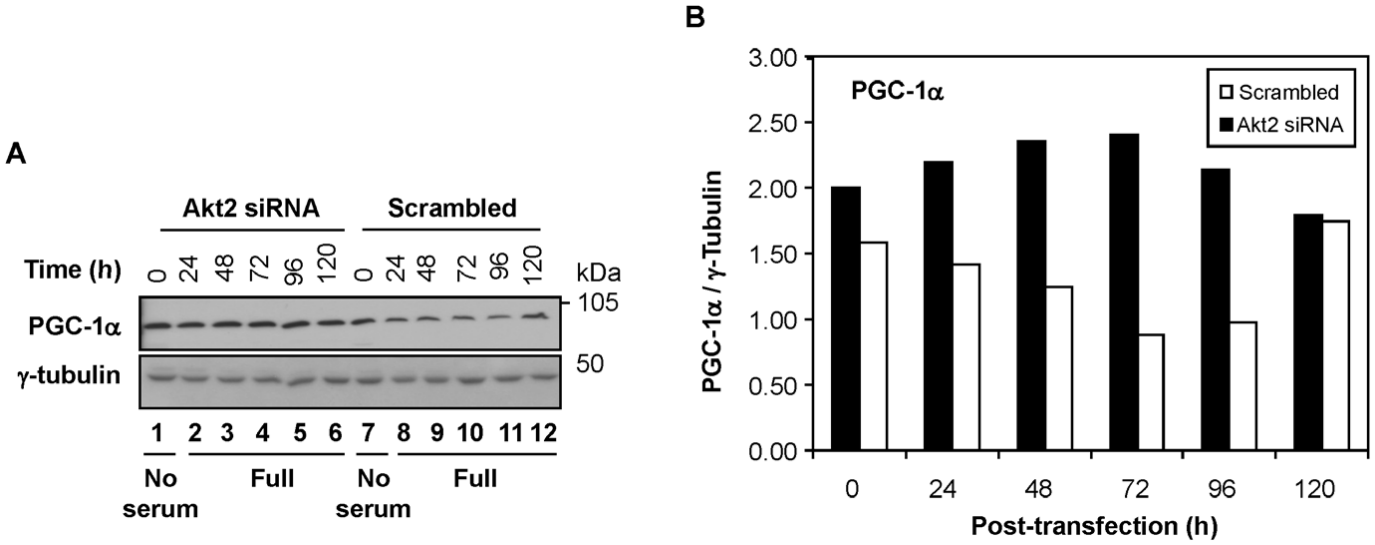
Akt2-ablation resulted in an increase of the PGC-1α protein level. (**A**) The level of PGC-1α was analyzed by Western blotting in MDA-MB231 cells transfected with Akt2 siRNA or scrambled siRNA. Data shown is representative of three independent trials. (**B**) The level of PGC-1α obtained by scanned gel images (in panel **A**) was analyzed by densitometry (Alpha Innotech). The intensity was normalized with that of γ-tubulin.

**Figure 7:**
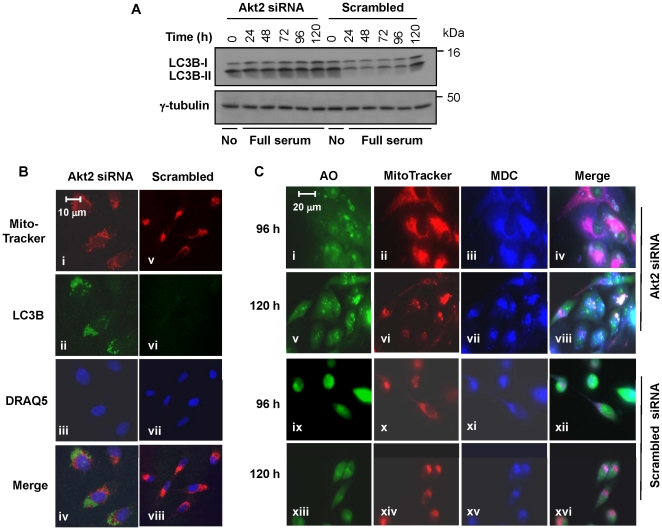
Akt2 ablation induced autophagy of mitochondria. (**A**) Akt2 ablation with siRNA resulted in an increase of LC3B-II. Western blotting was carried out with anti-LC3B antibody that recognizes both LC3B-I and LC3B-II. Cell extracts from Akt2-ablated or control cells (scrambled) were prepared at the timepoints indicated. “No” denotes no serum in the medium (i.e., Opti-MEM). Note the increased LC3B-II levels in the Akt2-ablated cells compared to the scrambled controls. (**B**) Immunofluorescent staining of LC3B was carried out at 96 h post-transfection. Red, green, and blue are MitoTracker (mitochondrial detection), LC3B (detection of LC3B, which is associated with autophagosomes), and DRAQ5 (nucleus detection), respectively. Akt2 siRNA and “Scrambled” denote MDA-MB231 cells transfected with Akt2 siRNA or scrambled siRNA, respectively. (**C**) Cells on a glass coverslip were transfected with Akt2 siRNA or scrambled siRNA, followed by triple-staining, first with MitoTracker Red, followed by acridine orange (AO) and monodansylcadaverine (MDC). Sampling timepoints after transfection are indicated left to panel.

With respect to mitochondrial biogenesis, the following two signaling mechanisms could be relevant to the phenotype shown in the Akt2-knockdown cells. First, insulin suppresses FoxO1 transcription in an Akt-dependent mechanism, resulting in the prevention of FoxO1 binding to PGC-1α, which in turn can inhibit the transcription of genes involved in gluconeogenesis [53]. However, there was no notable alteration in FoxO1 phosphorylation at Ser256 in the Akt2 knockdowns (Fig. S6A). Second, Li et al. [54] demonstrated that Akt2 phosphorylated PGC-1α at Ser570 could inhibit the transcription of genes involved in gluconeogenesis in a manner independent of FoxO1 in hepatocytes. Since an antibody specific for phosphorylated PGC-1α at the Ser570 residue was not yet available, we analyzed the level of PGC-1α proteins in Akt2-ablated cells as an alternative. As shown in Fig. 6, the level of PGC-1α was elevated in the Akt2-ablated cells at least up to 96 h post-transfection. This data suggests that Akt2 ablation resulted in an increase of mitochondrial biogenesis through the PGC-1α pathway. The level of PGC-1α decreased to the same level of the scrambled control by 120 h post-transfection, probably due to the start of mitochondrial autophagy (see Fig. 7 and relevant discussion below).

### Akt2-ablated MDA-MB231cells eventually died by mitochondrial autophagy

Cells may undergo autophagy when the mTOR pathway is blocked; the upregulation of p27 persists; or the downregulation of Cdk2 is prolonged [52], [55]. All of these abnormalities were observed in Akt2-ablated MDA-MB231 cells (Figs. 2–4), suggesting that Akt2-ablated cells may undergo autophagy. To test this hypothesis, we analyzed the level and migration of LC3B. LC3B-I is lipidated (i.e., phosphatidylethanolamine conjugated) during the activation of autophagy, which becomes associated with newly formed autophagosomes [56], [57]. Since the lipidated form (i.e., LC3B-II) migrates slightly fast on the gel, this can be a good marker for the activation of autophagy. As expected, the level of LC3B-II was substantially higher in Akt2-ablated cells than the control (Fig. 7A). This data is consistent with the idea that Akt2 ablation induces autophagy. In contrast, the levels of LC3B-II did not notably increase in cells transfected with Akt1 siRNA or Akt3 siRNA, compared to cells transfected with scrambled siRNA (Fig. S8).

Serum-starvation, rapamycin, and PI3K inhibitors (such as wortmannin and LY294002) are all inducers of autophagy. It should be noted that the level of LC3B-II is high in the cells maintained in serum-free medium for 24 h during the transfection period (Fig. 7, 0 h timepoint), suggesting that a substantial number of cells undergo autophagy under the serum starvation conditions, regardless of the Akt2 status. Thus, the 0 h timepoint can essentially function as a positive control for the activation of autophagy. The level of LC3B-II is also high at the 120 h timepoint in the control. This may be due to nutrient depletion in the growth medium since the cells at this timepoint were confluent (Fig. 7A, scrambled control, 120 h).

To confirm the activation of autophagy in Akt2-ablated cells, we examined the presence of autophagosomes using an immunostaining method with an anti-LC3B antibody, MitoTracker Red CMXRos, and DRAQ5 (nuclear staining). Data shown in Fig. 7B suggested that autophagosomes were co-localized with the mitochondria in cells transfected with Akt2 siRNA (Fig. 7B, iv), but not in the control (Fig. 7B, viii). In contrast, Akt1- or Akt3-ablated cells showed only the background level of autophagosomes (Fig. S8, iv, viii, and xii).

We further examined the induction of autophagy in Akt2-ablated cells using monodansylcadaverine (MDC), an autofluorescent compound shown to be incorporated into autophagolysosomes [58], [59]. In this experiment, cells were concurrently stained with acridine orange (AO) to observe the overall cell morphology and acidic vesicular organelles [60], and MitoTracker Red CMXRos to label mitochondria. Data from this experiment showed that the mitochondria were visibly enlarged and co-localized with MDC by 96 h post-transfection with Akt2 siRNA (Fig. 7C, compared panels ii–iv with x–xii). By 120 h post-transfection, the mitochondria in the Akt2-ablated cells were largely collapsed (Fig. 7C, compared panels vi–viii with xiv–xvi). Together, our data obtained from three different approaches suggest that the mitochondria are selectively targeted for autophagic degradation when the ablation ofAkt2 persists in MDA-MB231.

## Discussion

**Figure 8:**
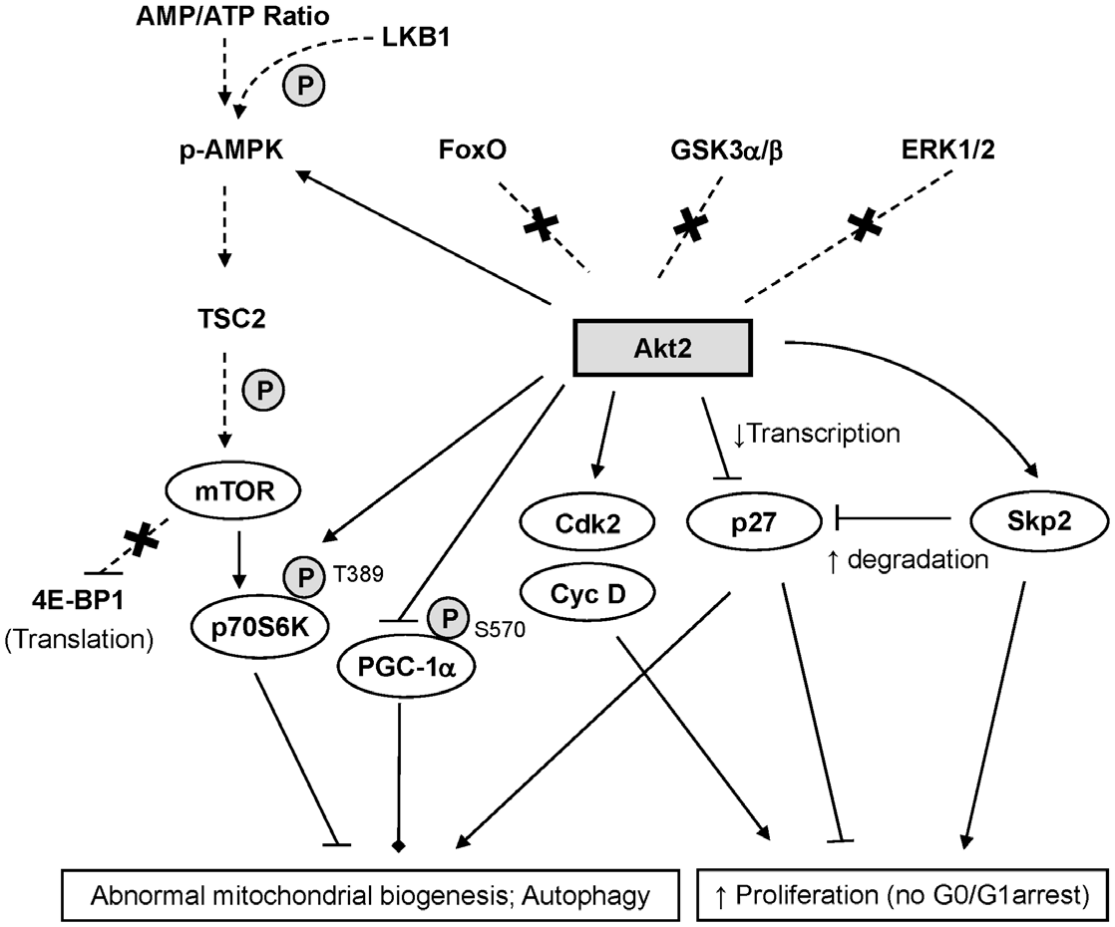
Summary of the Akt2-specific signal pathway and a proposed model. Akt2 facilitates cell cycle progression by regulating two separate pathways: (i) upregulation of Cdk2 and Cyclin D, and (ii) downregulation of p27 at the levels of transcription and protein stability through the upregulation of Skp2. In addition, Akt2 prevents abnormal mitochondrial biogenesis and pathological autophagy through the activation of p70S6K (i.e., phosphorylation at Thr389) and downregulation of PCG-1α. Broken lines and arrows are considered to be part of the pan-Akt pathway; however, our data suggests that Akt2 is not involved in the regulation of these signaling pathways. “×” denotes that these signaling pathways are not affected by Akt2 ablation. Solid lines are signaling pathways that have been confirmed by data presented in this paper. Arrows and “┴” denote up- and downregulation, respectively. ♦ denotes that this signal could cause abnormal mitochondrial biogenesis in the absence of Akt2.

Although Akt has been known to play an important role in cell proliferation and survival, the functional mechanism contributed by each isoform on these pathways is poorly understood. Using isoform-specific siRNA and the MDA-MB231 cell line as a breast cancer cell model, we examined the unique role of each Akt isoform on cell proliferation and survival. Our data demonstrates that Akt2 is the most critical player in the regulation of cell proliferation and survival among the three isoforms, at least in the breast cancer model examined (Fig. 1). Ablation of Akt2 promoted cell cycle arrest at G0/G1 through the downregulation of Cdk2 and cyclin D, and upregulation of p27, resulting in a decrease of cell proliferation. In addition, Akt2 ablation profoundly affected the mitochondria, initially increasing its volume and, eventually, causing cell death by mitophagy. As illustrated in Fig. 8, we found that Akt2 is not involved in the regulation of some of the known pan-Akt downstream substrates such as FoxO1, GSK-3α/β, and ERK1/2 in MDA-MB231 cells. The data presented in this paper lends further support to the growing lines of evidence in the literature that each Akt isoform regulates specific pathways.

### The role of Akt2 in the regulation of cell cycle progression and cell proliferation

While the rate of proliferation decreased in the cells transfected with Akt1- and Akt3-siRNA, cells void of Akt2 consistently showed the most pronounced decrease in proliferation among the three isoforms (Fig. 1C and Fig. S1C, D). This is, in part, due to the unique role of Akt2 in the promotion of cell cycle progression, as the ablation of Akt2 caused decreases in the levels of Cdk2 and cyclin D (Figs. 1–3). Cdk2 forms a complex with cyclin A or E, while cyclin D forms a complex with Cdk4 or Cdk6 [61]. Therefore, the downregulation of only two proteins (i.e., Cdk2 and cyclin D) in Akt2-ablated cells can ensure the effective arrest of the cell cycle in G0/G1 by simultaneously inactivating the Cdk2, 4 and 6 cell cycle engines.

The ablation of Akt1 did not notably alter the level of p27 (Fig. S6A), although it resulted in the downregulation of cyclin D (Fig. S4A). This data suggests that Akt1 also promotes cell cycle progression, but less effectively than Akt2 in MDA-MB231 cells. In contrast to Akt1 and Akt2, the ablation of Akt3 did not alter the level of Cdk2 (Fig. S3A), cyclin D (Fig. S4B), or p27 (Fig. S6B), suggesting that Akt3 is not directly involved in the regulation of cell cycle progression or proliferation in MDA-MB231 cells.

The upregulation of p27 in Akt2-ablated cells is another mechanism of effectively arresting cells in G0/G1 through the inactivation of Cdks [62]. We found that the upregulation of p27 in Akt2-ablated cells was mainly due to an increase in mRNA levels (Fig. S5C). However, p27 upregulation during the first 24 h after Akt2 ablation (i.e., 24 h post-transfection) may be due to the increase in protein stability (i.e., the 24 h timepoint in Fig. S5C; Fig. 3B, C). Since Skp2 was severely downregulated in Akt2-ablated cells at the same timepoint, this may have resulted in the stabilization of p27 protein (Fig. 3B). Our observation is consistent with published data that the level of p27 protein is regulated by both transcription and protein stability [63]–[65]. It should also be noted that p27 was mainly localized in the nucleus of Akt2-ablated cells, which can effectively downregulate Cdks (Fig. 3A). Along with the downregulation of Cdk2, cyclin D, and Skp2, the upregulation of p27 essentially guarantees the complete arrest of the cell cycle at G0/G1 in the Akt2-ablated cells.

Data presented here are largely consistent with numerous previous publications with respect to the role of Akt in facilitating cell cycle progression, cell proliferation, and the downregulation of p27, although many of them did not examine Akt in an isoform-specific manner [66]–[77]. However, our data is not consistent with that of Yang et al. [78]. Data obtained from experiments using constitutively active myristoylated Akt constructs, these authors concluded that both Akt1 and 2 efficiently inhibited the growth and proliferation of MDA-MB231 cells. The authors also concluded that Akt2 increased the stability and nuclear localization of p27, thus inhibiting cell cycle progression. Since these conclusions are completely opposite of what we describe in this paper, an important question arises: why are the conclusions by Yang et al. [79] and our group (and numerous other laboratories) completely different? We note that the experimental approach taken by Yang et al. [80] was very different from that of ours: they introduced constitutively active myristoylated Akt into the MDA-MB231 using an adenovirus-based vector, while our study used an siRNA-based approach under physiological conditions. It should be noted that cells may activate a negative feedback and/or cross-inhibitory control mechanism(s) under an extremely high level of constitutively active Akt [81]–[83]. Indeed, some of the data shown by Yang et al. [84] appear to support the activation of these negative control mechanisms, which might have resulted in shutting down the Akt and/or Erk signaling cascades. In addition, it is also important to note that Akt2 is localized at the mitochondria under physiological conditions [85]. It is presently unknown whether the Akt2-specific signaling is activated at the inner leaflet of the plasma membrane, similarly to the activation of Akt1. However, Yang et al. [86] assumed in their experiments that Akt2 is activated at the inner leaflet of the plasma membrane, which may or may not be correct. The constitutive activation of an extremely high level of myristoylated Akt2 at the plasma membrane is likely to alter the normal Akt and, possibly, other signaling pathways in the cell.

### Akt2 regulates p70S6K

The phosphorylation of p70S6K at Thr389 was almost completely downregulated in the Akt2-ablated MDA-MB231 cells, but not in the Akt1- or Akt3-ablated cells (Fig. 4A; Fig. S6). The finding that Akt2 signaling passes exclusively through the mTOR-p70S6K pathway was somewhat surprising, since published reports often speculated that the downstream signaling substrates of all three Akt isoforms were redundant. Clearly, our data does not support this previous suggestion. Consistent with our findings, Veilleux et al. [87] recently reported that the inhibition of mTORC1/S6K1 had little effect on Akt1 activation, but resulted in a significant decrease of Akt2 activity in 3T3-L1 adipocytes [88]. Moreover, the authors did not observe any alterations in p-AMPK when the function of mTORC1 was inhibited. Among many functions ascribed to the mTOR pathway, one of the most important functions is the regulation of protein synthesis. Akt2 is also known to regulate glucose transport and gluconeogenesis, and the inhibition of Akt2 function was associated with defects in glucose uptake [89]. Thus, our data is consistent with the idea that the Akt2-mTOR-p70S6K pathway is critical for cellular energy and metabolism that are directly relevant to cell proliferation and survival.

### Protection of mitochondrial autophagy by Akt2

One of the most exciting findings in the present study is the observation that the ablation of Akt2 had severe effects on the mitochondria of MDA-MB231 cells. The co-localization of Akt2 and the mitochondria from our previous study [90] provided the rationale to examine the mitochondria in the Akt2-ablated cells. The mitochondrial size and surface area increased substantially in the Akt2-ablated cells, suggesting that these cells had increased their mitochondrial content (Fig. 5). This finding was surprising since Akt2 ablation downregulated the protein synthesis pathway (Fig. 4). It was recently shown that signaling to PGC-1α may be regulated by the Akt2 pathway in a FoxO1-independent manner [91]. These authors demonstrated that phosphorylation of PGC-1α at Ser570 by Akt2 in hepatocytes inhibited the activation of PGC-1α and prevented the transcription of genes involved in gluconeogenesis [92]. Consistent with this previous finding, our data showed that the downregulation of Akt2 resulted in an increase of PGC-1α. Thus, this data validates the report of Li et al. [93], and further extends their findings by demonstrating that the deregulation is due to prolonged Akt2 inhibition.

One of the most critical consequences of Akt2 ablation is that it eventually leads a cell to pathological autophagy (Fig. 7). Most interestingly, the unique feature of this autophagy is that the mitochondria are the specific target (Fig. 7). However, this finding may not be unexpected since we have recently found that Akt2 is associated with the mitochondria [94]. An important conclusion derived from our study is that Akt2 wild type may be a critical protector of the mitochondria.

One interesting question is, how may an initial increase in the mitochondrial volume eventually lead to mitophagy? Our data suggests that Akt2 is involved in the regulation of mitochondrial biogenesis, as it apparently modulates PGC-1α (Fig. 6). Since the mTOR-p70S6K pathway is markedly downregulated (Fig. 4A), new protein synthesis is limited in Akt2-ablated cells. Under these conditions, the usage of proteins would have to be closely monitored. Uncontrolled mitochondrial biogenesis caused by the inactivation of the Akt2-mediated “surveillance” mechanism would result in an abnormal increase of the mitochondrial volume. This hypothesis is supported by the fact that PGC-1α was upregulated in Akt2-ablated cells (Fig. 6). Since uncontrolled “growth” of the mitochondria occurs while protein synthesis is severely limited due to the downregulation of p70S6K, the high activity of mitochondrial biogenesis cannot be sustainable for an extended duration in the Akt2-ablated cells. Thus, the deregulation of mitochondrial biogenesis in Akt2-ablated cells may eventually lead to the collapse of the entire mitochondrial homeostasis system (and may start to salvage the mitochondria to increase survivability). Hickson-Bick et al. [95] observed a similar phenomenon when PGC-1 was stimulated with lipopolysaccarides. We found that the ablation of neither Akt1 nor Akt3 resulted in mitophagy (Fig. S8). This may be relevant to the fact that, unlike Akt2 which is associated with the mitochondria, Akt1 and 3 are localized mainly to the cytoplasm and the nucleus, respectively [96].

In summary, among the three Akt isoforms, Akt2 is most relevant for cell proliferation and survival in MDA-MB231 cells. The former is achieved by the upregulation of Cdk2, cyclin D, and Skp2 as well as the downregulation of p27, while the latter is achieved by the activation of p70S6K and the modulation of PGC-1α. Since the downregulation of p27 is also relevant to cell survival [52], p27 is a key control point in the Akt2-mediated cell regulation. In terms of cancer therapy, selective inhibitors for the Akt isoforms have been developed but have not yet reached clinical trials (reviewed in [97]). Clinical trials with rapamycin have been shown to be effective in some, but not all cancers. Further, Arboleda et al. [98] found that Akt2 overexpression induced invasion and metastasis in breast and ovarian tumors. Thus, the data presented in this paper provide grounds that Akt2 can be one of the most promising targets for anti-cancer therapies, at least for breast cancer.

## Materials and Methods

### Cell Culture

The MDA-MB231 breast cancer cell line, purchased from ATCC (Manassas, VA), was grown in RPMI-1640 medium containing 10% (v/v) fetal bovine serum (Hyclone), 2.05 mM L-glutamine, 100 µg/ml streptomycin and 100 units/ml penicillin. Cells were maintained at 37°C in a humidified atmosphere with 5% CO_2_ and 95% air.

### Antibodies, Chemicals, and Reagents

Antibodies against the following proteins were purchased from Cell Signaling Technology (Danvers, MA): Akt1 (2H10), p-FoxO1(S256), FoxO1, p-GSK-3α/β (S9/S21), GSK-3β, p-p70S6K (T389), p70S6K, p-4E-BP1, 4E-BP1, p-AMPKα (T172), AMPKα, and Skp2. Antibodies against the following proteins were from Santa Cruz Biotechnology (Santa Cruz, CA): Akt2 (F7), Cyclin A, Cyclin D1, Cyclin E, p-ERK1/2 (p42/p44), p-p27 (T187), p27, and PGC-1α. Anti-phospho-p27 Thr157 and -Thr198 antibodies were from R&D Systems (Minneapolis, MN). Anti-Cdk2 and -Akt3 antibodies were purchased from Lab Vision Corporation (Fremont, CA) and Upstate Biotechnology (Lake Placid, NY), respectively. Anti-mouse, -rabbit, and -goat peroxidase-conjugated secondary antibodies were obtained from Sigma, Pierce, and Calbiochem, respectively. Fluorescein (FITC)-conjugated AffiniPure donkey and anti-mouse IgG secondary antibody used in the immunofluorescence experiments were from Jackson Immunoresearch (West Grove, PA). Anti-γ-tubulin antibodies, propidium iodide (PI), thiazolyl blue tetrazolium bromide (MTT), dimethyl sulfoxide (DMSO), acridine orange, and monodansylcadaverine (MDC) were purchased from Sigma. Lipofectamine 2000 transfection reagent, Opti-MEM I transfection media, DNAse I, and M-MLV were from Invitrogen. MitoTracker Red CMXRos Mitochondrial Probe was from Cambrex Bioscience Walkersville, Inc. (Charles City, Iowa). Methylene blue was from Acros Organics (Fisher).

### Small interfering RNA (siRNA)

Cells (∼30% confluence) were plated 24 h prior to transfection with siRNA in antibiotic-free medium. Transfections were carried out using Lipofectamine 2000 (Invitrogen) according to the manufacturer's protocol. Transfection was carried out in Opti-MEM Reduced Serum Medium (Invitrogen) for 24 h. The 0 h timepoint described throughout this manuscript is defined as the end of the transfection procedure (i.e., 24 h after transfection was initiated). The “Reduced Serum Medium” does not actually contain any FBS (hence, denoted as “no serum” in this paper). At the 0 h timepoint, the medium was replaced with RPMI 1640 supplemented with 10% FBS (Hyclone) in all subsequent timepoints (24–120 h), which is defined as complete medium. To knockdown each Akt isoform, two different siRNA oligonucleotides for each Akt isoform were used: (A) siRNA set 1: Akt1 (5′ GGCUCCCCUCAACAACUUC 3′), Akt2 (5′ GGAUGAAGUCGCUCACACA 3′), and Akt3 (5′ GGACCGCACACGUUUCUAU 3′). (B) siRNA set 2: Akt1 (5′ GCCCUCAGAACAAUCCGAU 3′), Akt2 (5′GGUCGACACAAGGUACUUC 3′), and Akt3 (5′ GGCCAAGAUACUUCCUUUU 3′).

Control samples were transfected with scrambled siRNA (Scrambled Control, 10 nM) or transfected with reagent alone (Reagent Control). All siRNA oligonucleotides, including scrambled controls, were purchased from Ambion.

### Cell proliferation and survival assays

Cell proliferation was measured using an MTT assay and BrdU incorporation. For the MTT assay, cells were transfected with siRNA specific to each Akt isoform in 10 cm plates (Sarstedt). At the 24 h timepoint after transfection was initiated (i.e., 0 h timepoint), cells were trypsinized, washed, and counted. Three thousand cells (in 100 µl culture medium) were plated in a well of a 96-well clustered dish, and maintained until samples were taken at scheduled timepoints (48–120 h post-transfection). A portion of the leftover cells at 24 h (i.e., 0 h timepoint described in this paper) was used for Western blot analysis to confirm the efficiency of the siRNA transfection. At each timepoint, 10 µl of MTT stock (5 mg/ml MTT in PBS) was added to each well and incubated for 4 h at 37°C, 5% CO_2_. The MTT-culture medium was gently removed from the well using a pipette. 200 µl of DMSO was added to each well and mixed thoroughly, followed by analysis using a SPECTRAmax 340 PC plate reader (Molecular Devices). Absorbance was measured at 570 nm (and 650 nm as reference wavelength). All absorbance measurements were corrected to the reference wavelength (Corrected absorbance = 570–650 nm).

5-Bromo-2′-deoxy-uridine (BrdU) incorporation was carried out according to the manufacturer's protocol (Roche). Briefly, MDA-MB231 cells were plated on a 1.8-cm (0.13–0.17-mm thick) sterile glass-coverslip (Fisher) that had been placed in the 6-well plate 24 h prior to transfection. Cells on the coverslip were transfected with siRNA, followed by analysis of BrdU incorporation at 0, 24, and 48 h post-transfection. BrdU pulse-labelling was carried out during the last 45 min of each sampling timepoint. A minimum of 10 fields per coverslip were documented using an LSM 510 Meta laser scanning confocal microscope (Carl Zeiss) with a 488-nm laser (filter settings 505–530 nm).

For the colony forming assay, cells harvested at 24 h post-siRNA transfection were plated on a 10-cm dish (∼1000 cells). After 10–12 days of incubation, the culture medium was removed, and the plates were gently washed with PBS. Cells were then fixed and stained with 2% (w/v) methylene blue in 100% methanol for 10 min at room temperature. Colonies comprised of >50 cells were counted as positives.

### Immunostaining

Cells were plated on a 1.8-cm sterile glass coverslip that had been placed in a well of a 6-well clustered dish. Cells on the coverslip were transfected after overnight incubation. At a scheduled timepoint, cells were washed with PBS, fixed with 4% paraformaldehyde for 10 min, and then permeabilized in 0.2% Triton X-100 for 5 min. The coverslips were treated with blocking solution (10% horse serum, 1% BSA, 0.02% NaN_3_, in PBS) for 1 h at room temperature, followed by immunostaining with an appropriate antibody. Primary antibodies (diluted in 1% BSA in TBS) were incubated for 45 min at the following concentrations: Akt2 (1∶10), Cdk2 (1∶20), and p27 (1∶10), and then washed in TBS. A fluorescein (FITC)-conjugated donkey anti-mouse IgG secondary antibody was diluted 1∶100 (1% BSA in TBS), and incubated for 30 min. The coverslip was washed again in TBS, and mounted in 90% glycerol/PBS. For double-labelling of Akt2 and MitoTracker, cells were first incubated with 50 nM MitoTracker Red CMXRos in cell culture media for 30–45 min (37°C, 5% CO_2_), followed by fixation and permeabilization prior to immunostaining with an anti-Akt2 antibody. Immunostained cells were then observed by confocal microscopy (LSM 510 Meta laser scanning microscope, Carl Zeiss) using a 63× objective lens and two laser lines for excitation with the following band pass settings: Argon 488 nm (band pass 505–530 nm), HeNe 543 nm (long pass 560 nm). Images were captured using an LSM 510 Meta confocal microscope and associated software. Mitochondrial surface and total cell areas were calculated using the same software. The relative mitochondrial surface area was calculated as follows: [(mitochondrial surface area/total cell surface area of a cell)] (in µm^2^).

### Quantitative PCR (Q-PCR)

Total RNA was extracted from cells using an RNeasy kit (Qiagen) according to the manufacturer's protocol. The concentration of RNA was measured using a spectrophotometer (UV 1101 Biotech Photometer) and the integrity was verified by agarose gel (1%) electrophoresis. One microgram of DNAse I-treated RNA was reverse transcribed with M-MLV (Invitrogen) and 10 mM dNTPs (IB Labs). Q-PCR was carried out as described previously [99] using an ABI Prism 7900 HT Sequence Detection System (SDS) (Applied Biosystems). The PCR conditions were as follows: 95°C for 15 s, 55°C for 15 s, and 72°C 30 s, for 40 cycles. The RNA isolated from the control was used to calibrate the standard curve. The mRNA levels were calculated by comparing the ratio of Akt2:S28 and p27:S28. The following primers were used for PCR reactions (300 nM): Akt2 forward 5′-CAAGGATGAAGTCGCTCACACA-3′ and Akt2 reverse 5′-GAACGGGTGCCTGGTGTTC-3′; p27 forward 5′-CGGTGGACCACGAAGAGTTAA-3′ and p27 reverse 5′-GGCTCGCCTCTTCCATGTC-3′. Primer sequences for the S28 housekeeping gene were as follows: S28 forward 5′-TCCATCATCCGCAATGTAAAAG-3′ and S28 reverse 5′-GCTTCTCGCTCTGACTCCAAA-3′. Data was acquired and analyzed using ABI SDS software (version 2.1) (Applied Biosystems).

### Detection of autophagy

Cells (transfected with siRNA) were incubated with 50 nM MitoTracker Red CMXRos in culture media for 30 min in a cell culture incubator, followed by treatment with acridine orange (1.0 µg/ml) and MDC (a specific marker for the autophagosome) (0.05 mM) as described previously [58], [60], [100], [101]. Cells were then rinsed with PBS and mounted on a glass slide for visualization by fluorescent microscopy. Fluorescent images were captured with an LSM 510 Meta confocal microscope (Carl Zeiss) using a 63× objective and three laser lines for excitation with the following band pass filter settings used for detection: Argon 488 (band pass 505–530 nm), HeNe 543 nm (long pass 560) and 633 nm (long pass 650).

To determine the process of autophagy, the induction of LC3B was examined in cells transfected with Akt2 or scrambled siRNA. At 96 h post transfection, cells were washed in PBS, fixed in 4% paraformaldehyde, and permeabilized with 100% (v/v) ice-cold methanol for 10 min at −20°C. The cells on coverslips were then treated with blocking solution for 1 h at room temperature. The primary antibody (LC3B) was diluted in TBS containing 1% BSA prior to adding to the cells, which was incubated overnight at 4°C. The next day, the cells were washed and incubated with secondary antibody. Finally, cells were rinsed and counterstained with DRAQ5 (10 µm) for 30 min at room temperature (Biostatus Ltd., Shepshed, Leicestershire, UK), prior to visualization by confocal microscopy.

### Statistical analysis

A univariate 2-way ANOVA with subsequent post-hoc analysis for significance testing (Tukey p<0.05) was used to determine differences in cellular proliferation and BrdU incorporation, as well as in the analyses of mitochondrial surface area. Univariate analysis of variance (One-way ANOVA) was carried out to analyze colony formation ability. All statistics were performed using SPSS v.12.0 for Windows.

## Supporting Information

Figure S1 Akt2 ablation resulted in a decrease of cell proliferation and survival. (A). Western blotting was carried out with samples isolated from MDA-MB231 cells transfected with isoform-specific siRNA Set #2 alone or in combination with other siRNA oligos as indicated. “R” denotes cells transfected with reagent alone (i.e., a mock transfection control). Akt1–3 at the left of the panel denotes antibodies against Akt1–3 proteins, respectively. γ-tubulin was used as a loading control. Data shown is representative of four independent experiments. (B) Colony-forming assay with siRNA Set #2. As with the Set #1 sample, siRNA targeted to Akt2 caused the greatest decrease in colony forming ability. Results shown are an average of four independent trials. Data are expressed as the average mean colony formation, and error bars are standard error.
(0.79 MB TIF)

Figure S2 Cells transfected with Akt2 siRNA were arrested at G0/G1 phase. MDA-MB231 cells were transfected with Akt2 siRNA, and their cell cycle positions were determined by flow cytometry. 0 h, 24 h and 48 h on top of the panels are time (h) post-transfection (for 24 h in Opti-MEM which does not contain FBS). “Reagent” and “Controls” are mock transfection and “kept-in-incubator” controls, respectively. 2n and 4n are two (i.e., G1 phase) and four (G2/M phase) chromosomal contents, respectively.
(0.54 MB TIF)

Figure S3 Cdk2 is downregulated in Akt2-, but not Akt1- and Akt3-ablated cells. (A) Western blot assays with anti-Cdk2 or -γ-tubulin (loading control) antibodies were carried out with MDA-MB231 cells transfected with siRNA specific to Akt1, 2, 3, or in combination. The sampling time was 24 h post-transfection. “R” is as per legend to Figure 1. “C” denotes a control kept in the incubator during the transfection process. (Lower panel) The relative level of Cdk shown in the upper panel was quantified by densitometry and normalized with loading control. (B) The relative level of Cdk2 measured in a timecourse manner (0–120 h post-transfection) in cells transfected with a second set of Akt2 siRNA oligo (Set #1). Data was generated by densitometry from Western blot. Data shown is from three independent experiments. The relative levels of cyclin D1 (C) and cyclin E (D) quantified with densitometry and normalized to the loading control. Error bars are standard errors.
(0.70 MB TIF)

Figure S4 Effects of Akt 1 or Akt3 ablation on the level of cyclin D. (A) Akt1 ablation in MDA-MB231 cells resulted in the downregulation of cyclin D. (B) Ablation of Akt3 in MDA-MB231 cells resulted in little change in the level of cyclin D.
(1.09 MB TIF)

Figure S5 Levels of p27 phosphorylation at Thr157 (A) and Thr198 (B) are shown. Shown is the Q-PCR-based analysis of p27 mRNA level (C) and Akt2 mRNA level (D). Data are results of three independent experiments (in triplicate for each experiment), and were normalized to the value of tubulin (for protein) or S28 mRNA (for mRNA). Error bars are standard errors.
(0.59 MB TIF)

Figure S6 The effects of Akt 1 and 3 on p27 and p70S6K. (A) Akt1 ablation in MDA-MB231 cells did not affect the levels of p27 protein and p70S6K phosphorylation. (B) Ablation of Akt3 in MDA-MB231 cells resulted in little changes in the level of phosphorylation of p70S6K.
(1.30 MB TIF)

Figure S7 Examination of the downstream Akt pathways in MDA-MB231 cells transfected with Akt2 siRNA or scrambled siRNA. (A, B) Western blot analyses were carried out with antibodies against proteins listed left to panels. γ-tubulin was used as loading control. Data shown are representative of three independent experiments (p-ERK was performed in duplicate). No serum, Opti-MEM transfection medium only; Full, complete medium containing 10% FBS.
(1.24 MB TIF)

Figure S8 Neither the ablation of Akt1 nor Akt3 induced substantial mitophagy. (A) Knockdown of Akt1 (left panel) and Akt3 (right panel) with siRNA did not increase substantially the amount of LC3B-II. Western blotting was carried out with anti-LC3B antibody that recognizes both LC3B-I and LC3B-II. The extracts from Akt2-ablated or control cells (scrambled) were prepared at timepoints indicated. “No” denotes no serum in the medium (i.e., Opti-MEM). (B) Cells were transfected with Akt1 siRNA (i–iv), Akt3 siRNA (v–viii), or scrambled siRNA (ix–xii), followed by immunofluorescent staining. Red, green, and blue are MitoTracker (mitochondrial detection), LC3B (mostly associated with autophagosomes), and DRAQ5 (nucleus detection), respectively.
(2.69 MB TIF)
