# Supplementary figures and images for: Ablation of Akt2 Induces Autophagy through Cell Cycle Arrest, the Downregulation of p70S6K, and the Deregulation of Mitochondria in MDA-MB231 Cells
Source: PLoS One. 2011 Jan 31;6(1):e14614. doi: 10.1371/journal.pone.0014614 (PMC3031501; doi:10.1371/journal.pone.0014614)

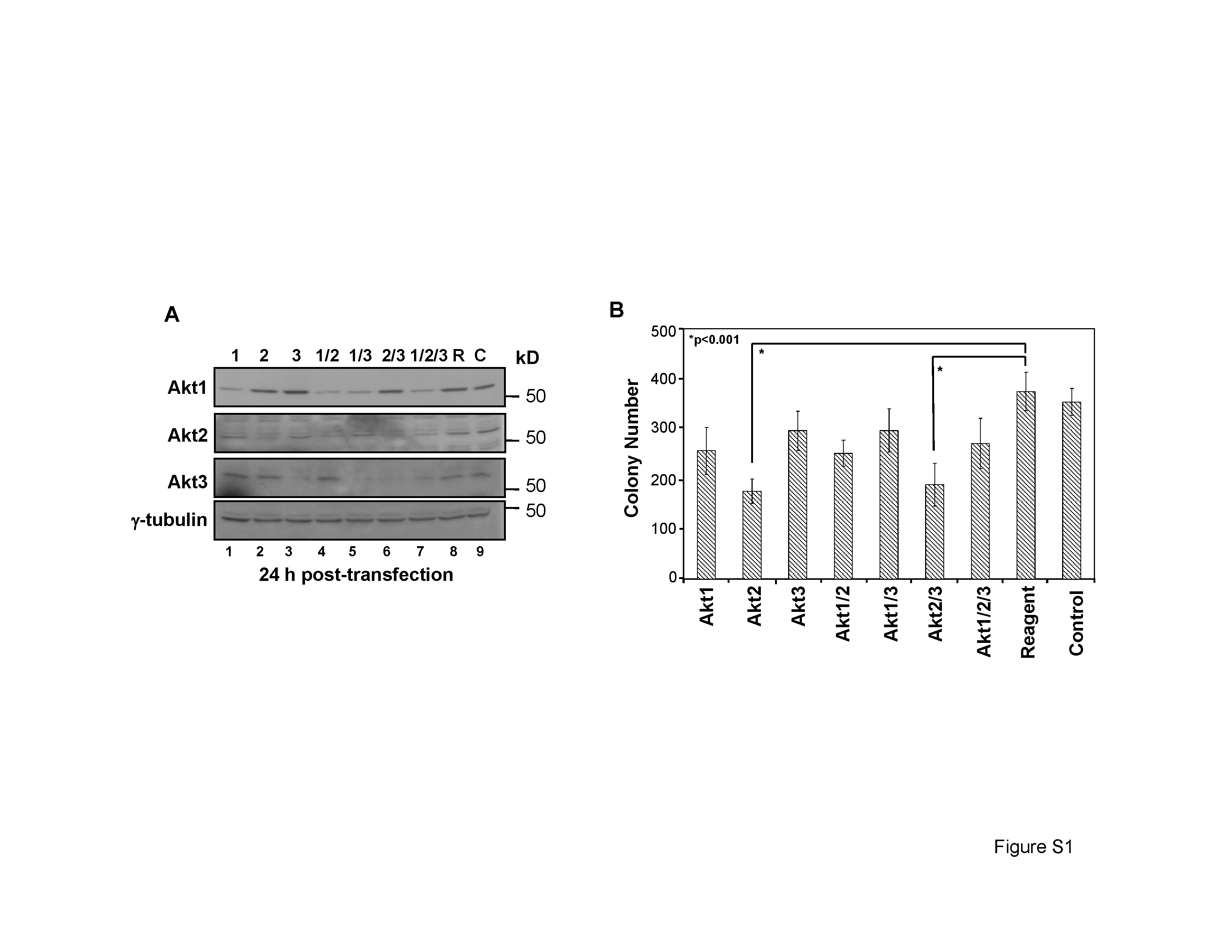

Supplement: Figure S1 — Akt2 ablation resulted in a decrease of cell proliferation and survival. (A). Western blotting was carried out with samples isolated from MDA-MB231 cells transfected with isoform-specific siRNA Set #2 alone or in combination with other siRNA oligos as indicated. “R” denotes cells transfected with reagent alone (i.e., a mock transfection control). Akt1–3 at the left of the panel denotes antibodies against Akt1–3 proteins, respectively. γ-tubulin was used as a loading control. Data shown is representative of four independent experiments. (B) Colony-forming assay with siRNA Set #2. As with the Set #1 sample, siRNA targeted to Akt2 caused the greatest decrease in colony forming ability. Results shown are an average of four independent trials. Data are expressed as the average mean colony formation, and error bars are standard error. (0.79 MB TIF) [file pone.0014614.s001.tif]

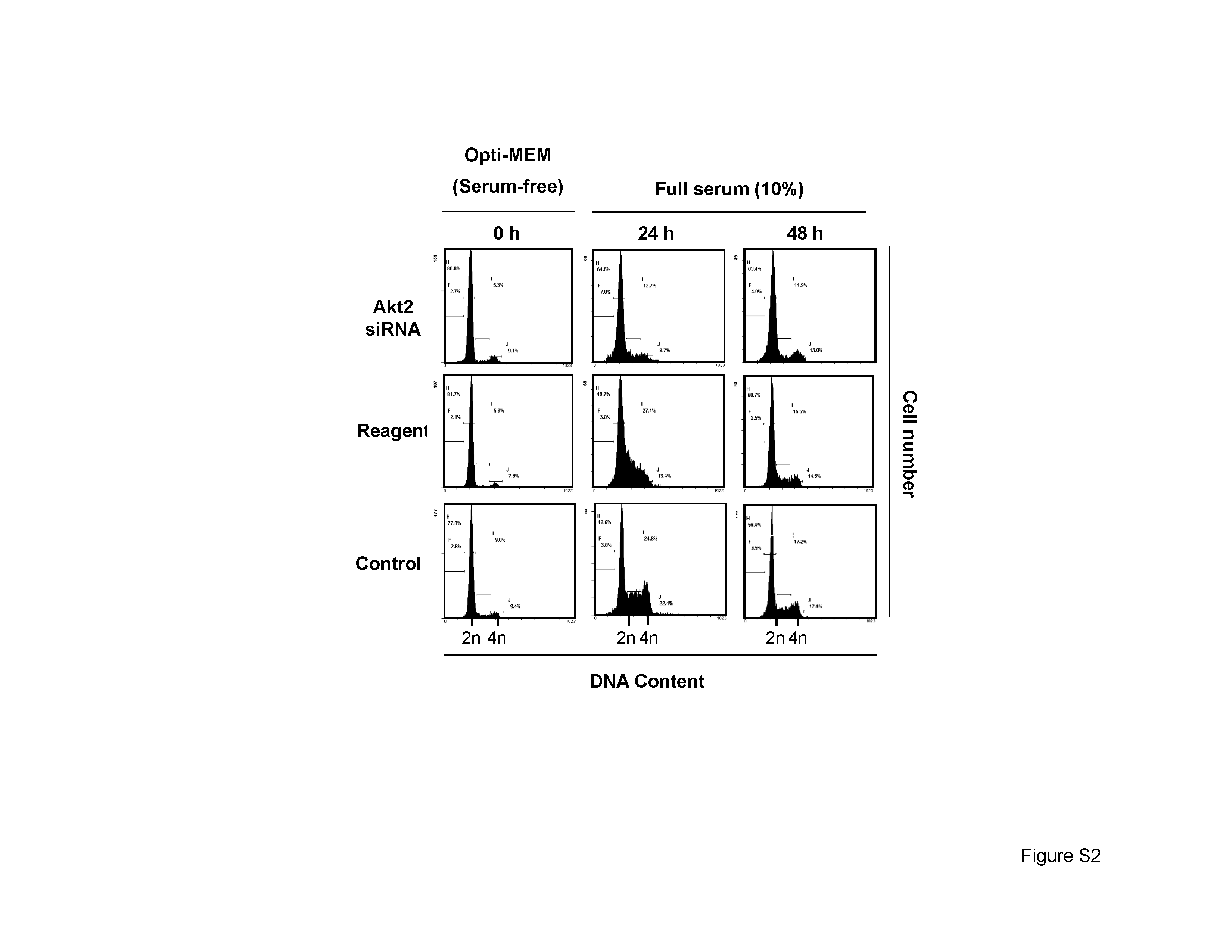

Supplement: Figure S2 — Cells transfected with Akt2 siRNA were arrested at G0/G1 phase. MDA-MB231 cells were transfected with Akt2 siRNA, and their cell cycle positions were determined by flow cytometry. 0 h, 24 h and 48 h on top of the panels are time (h) post-transfection (for 24 h in Opti-MEM which does not contain FBS). “Reagent” and “Controls” are mock transfection and “kept-in-incubator” controls, respectively. 2n and 4n are two (i.e., G1 phase) and four (G2/M phase) chromosomal contents, respectively. (0.54 MB TIF) [file pone.0014614.s002.tif]

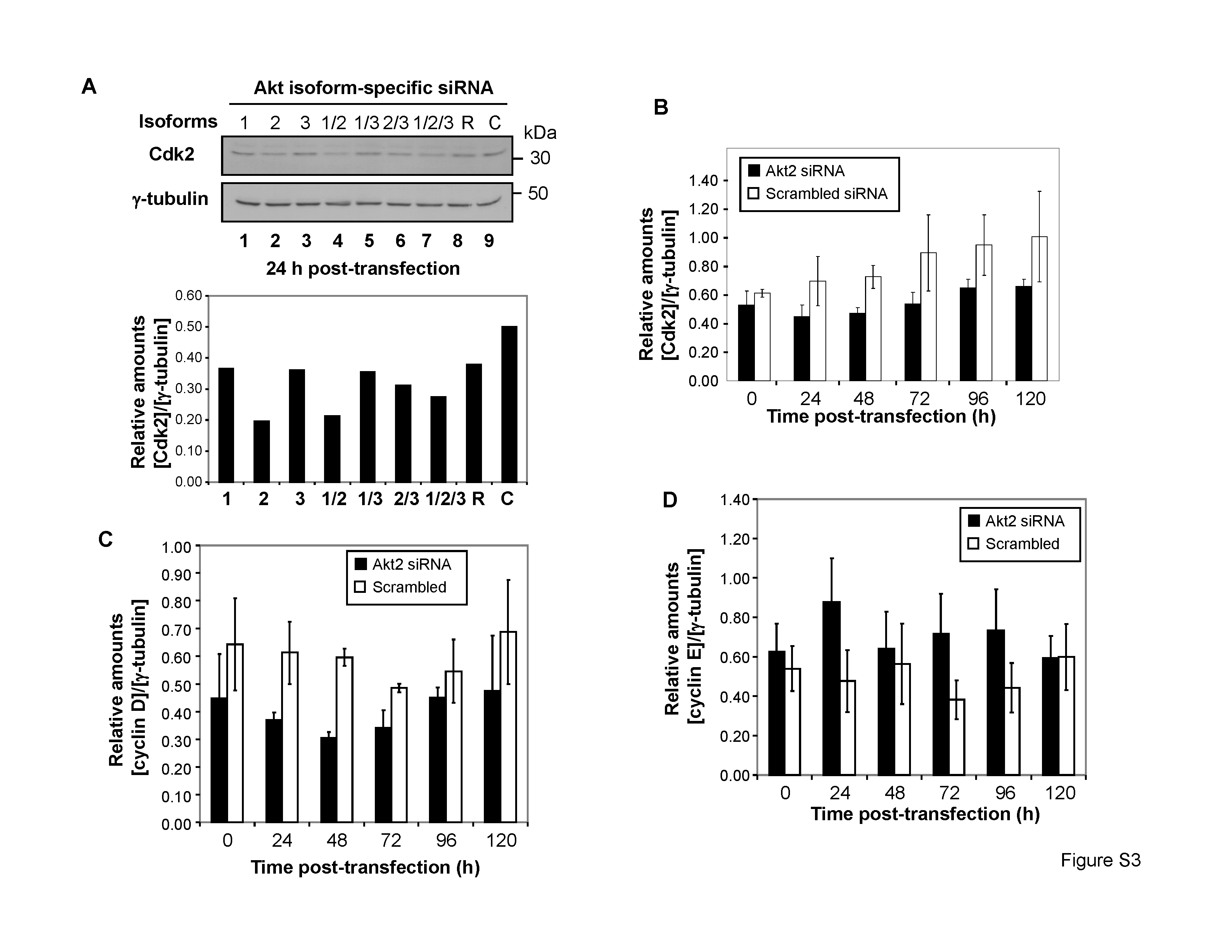

Supplement: Figure S3 — Cdk2 is downregulated in Akt2-, but not Akt1- and Akt3-ablated cells. (A) Western blot assays with anti-Cdk2 or -γ-tubulin (loading control) antibodies were carried out with MDA-MB231 cells transfected with siRNA specific to Akt1, 2, 3, or in combination. The sampling time was 24 h post-transfection. “R” is as per legend to Figure 1. “C” denotes a control kept in the incubator during the transfection process. (Lower panel) The relative level of Cdk shown in the upper panel was quantified by densitometry and normalized with loading control. (B) The relative level of Cdk2 measured in a timecourse manner (0–120 h post-transfection) in cells transfected with a second set of Akt2 siRNA oligo (Set #1). Data was generated by densitometry from Western blot. Data shown is from three independent experiments. The relative levels of cyclin D1 (C) and cyclin E (D) quantified with densitometry and normalized to the loading control. Error bars are standard errors. (0.70 MB TIF) [file pone.0014614.s003.tif]

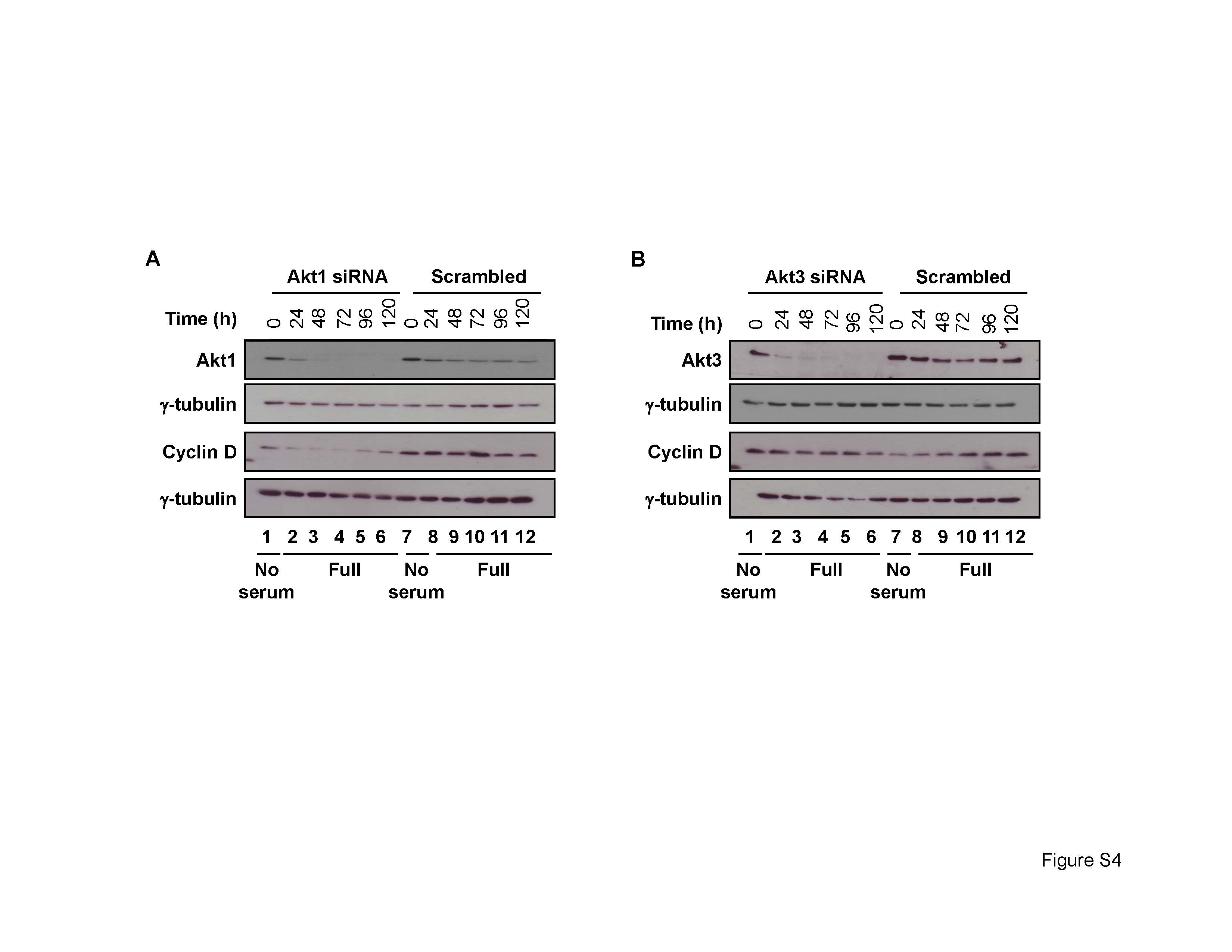

Supplement: Figure S4 — Effects of Akt 1 or Akt3 ablation on the level of cyclin D. (A) Akt1 ablation in MDA-MB231 cells resulted in the downregulation of cyclin D. (B) Ablation of Akt3 in MDA-MB231 cells resulted in little change in the level of cyclin D. (1.09 MB TIF) [file pone.0014614.s004.tif]

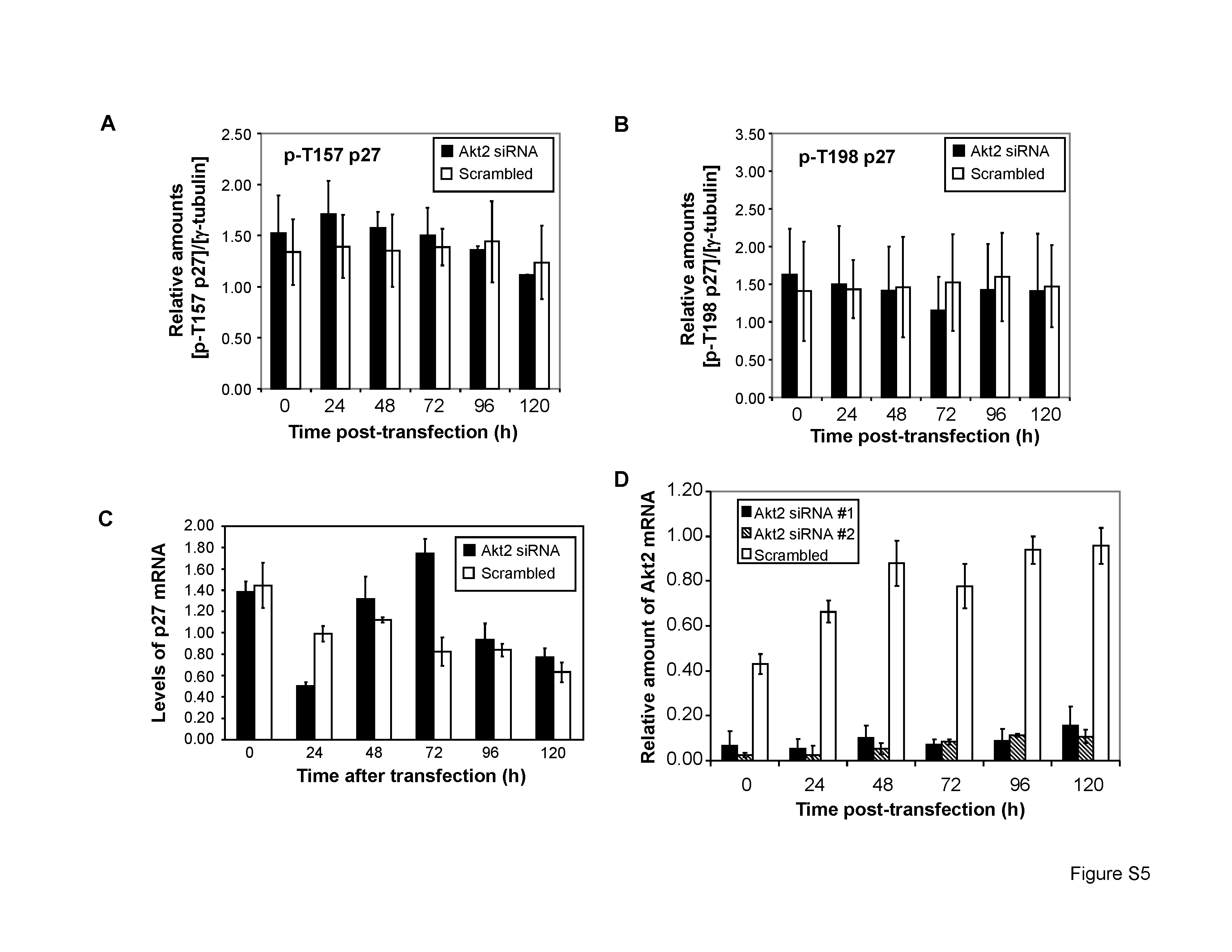

Supplement: Figure S5 — Levels of p27 phosphorylation at Thr157 (A) and Thr198 (B) are shown. Shown is the Q-PCR-based analysis of p27 mRNA level (C) and Akt2 mRNA level (D). Data are results of three independent experiments (in triplicate for each experiment), and were normalized to the value of tubulin (for protein) or S28 mRNA (for mRNA). Error bars are standard errors. (0.59 MB TIF) [file pone.0014614.s005.tif]

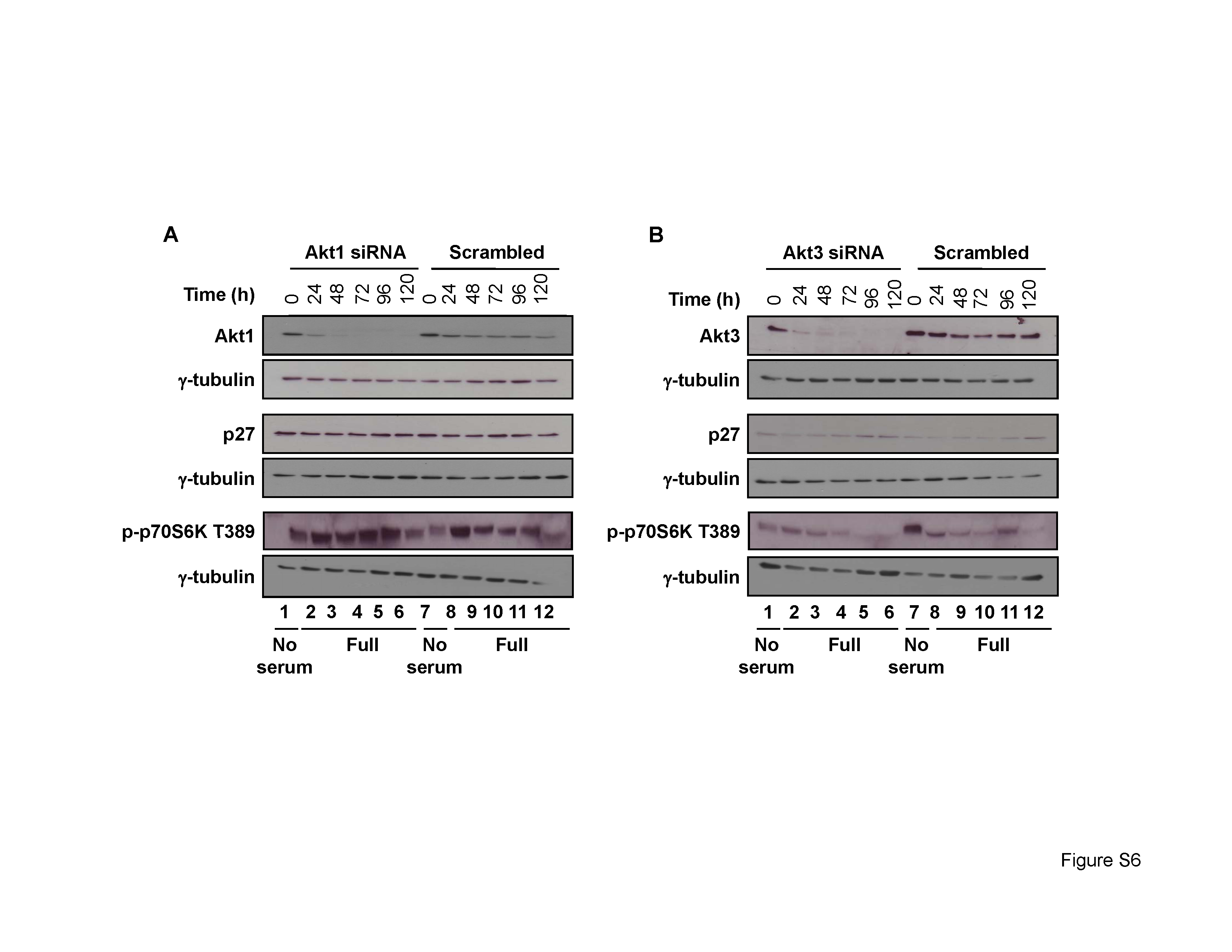

Supplement: Figure S6 — The effects of Akt 1 and 3 on p27 and p70S6K. (A) Akt1 ablation in MDA-MB231 cells did not affect the levels of p27 protein and p70S6K phosphorylation. (B) Ablation of Akt3 in MDA-MB231 cells resulted in little changes in the level of phosphorylation of p70S6K. (1.30 MB TIF) [file pone.0014614.s006.tif]

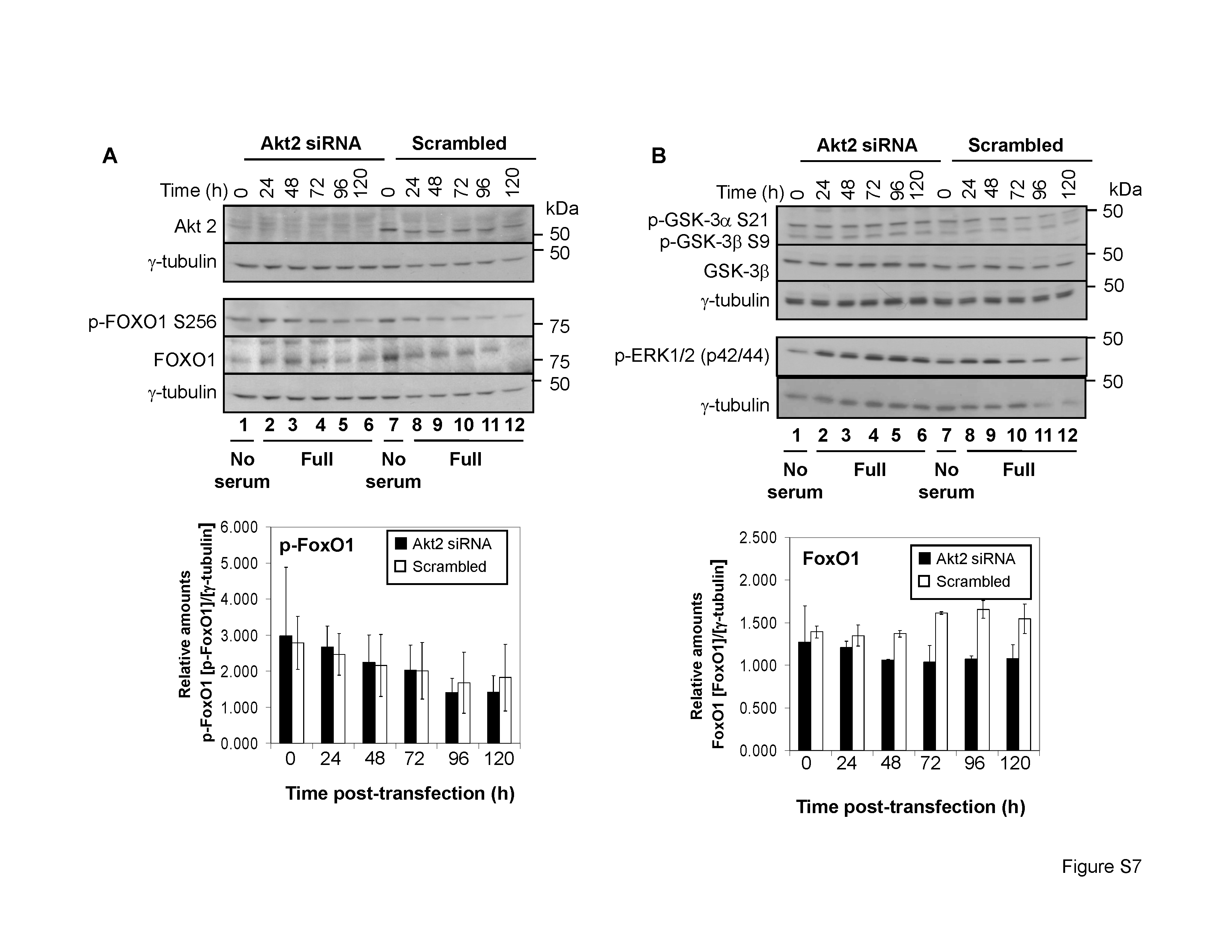

Supplement: Figure S7 — Examination of the downstream Akt pathways in MDA-MB231 cells transfected with Akt2 siRNA or scrambled siRNA. (A, B) Western blot analyses were carried out with antibodies against proteins listed left to panels. γ-tubulin was used as loading control. Data shown are representative of three independent experiments (p-ERK was performed in duplicate). No serum, Opti-MEM transfection medium only; Full, complete medium containing 10% FBS. (1.24 MB TIF) [file pone.0014614.s007.tif]

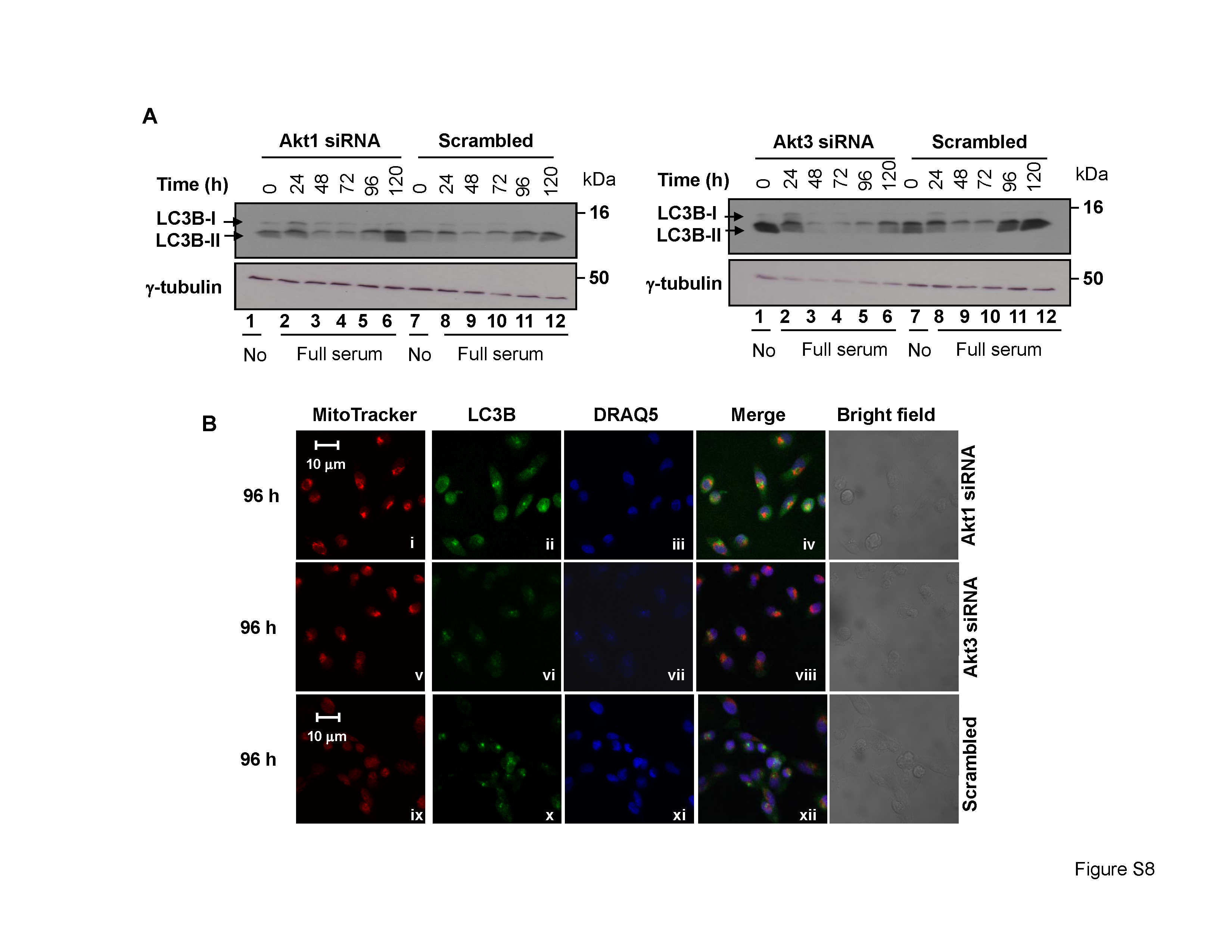

Supplement: Figure S8 — Neither the ablation of Akt1 nor Akt3 induced substantial mitophagy. (A) Knockdown of Akt1 (left panel) and Akt3 (right panel) with siRNA did not increase substantially the amount of LC3B-II. Western blotting was carried out with anti-LC3B antibody that recognizes both LC3B-I and LC3B-II. The extracts from Akt2-ablated or control cells (scrambled) were prepared at timepoints indicated. “No” denotes no serum in the medium (i.e., Opti-MEM). (B) Cells were transfected with Akt1 siRNA (i–iv), Akt3 siRNA (v–viii), or scrambled siRNA (ix–xii), followed by immunofluorescent staining. Red, green, and blue are MitoTracker (mitochondrial detection), LC3B (mostly associated with autophagosomes), and DRAQ5 (nucleus detection), respectively. (2.69 MB TIF) [file pone.0014614.s008.tif]
